# Design and Structural Requirements of the Potent and Safe TLR-9 Agonistic Immunomodulator MGN1703

**DOI:** 10.1089/nat.2015.0533

**Published:** 2015-06-01

**Authors:** Manuel Schmidt, Nicole Hagner, Alberto Marco, Sven A. König-Merediz, Matthias Schroff, Burghardt Wittig

**Affiliations:** ^1^ Mologen AG, Berlin, Germany.; ^2^ Foundation Institute Molecular Biology and Bioinformatics, Freie Universitaet, Berlin, Germany.; ^3^ Department of Animal Health and Anatomy, Universidad Autonoma de Barcelona, Barcelona, Spain.; ^4^ Vivotecnia, Madrid, Spain.

## Abstract

Single-stranded oligodeoxynucleotides (ODN), containing nonmethylated cytosine–guanine motifs (CpG ODN), are recognized by the innate immune system as “danger signals.” CpG ODN are efficacious immunomodulators but require phosphorothioate (PT) or other backbone modifications for metabolic stability, which cause toxicities in mice and primates. We therefore designed a covalently closed DNA molecule (dSLIM^®^) where two single-stranded loops containing CG motifs are connected through a double-stranded stem in the absence of any nonnatural DNA component. The most promising immunomodulator, MGN1703, comprises two loops of 30 nucleotides containing three CG motifs each, and a connecting stem stem of 28 base pairs. MGN1703 stimulates cytokine secretion [interferon (IFN)-α, IFN-γ, interleukin (IL)-12, IL-6, and IL-2] and activates immune cells by increased expression of CD80, CD40, human leukocyte antigen (HLA)-DR and ICAM-1. Efficacy of immunomodulation strictly depends on the descriptive dumbbell shape and size of the molecule. Variations in stem length and loop size lead to reduced potency of the respective members of the dSLIM^®^ class. In a representative mouse model, toxicities from injections of high amounts of a CpG ODN-PT and of MGN1703 were evaluated. The CpG ODN-PT group showed severe organ damage, whereas no such or other pathologies were found in the MGN1703 group. Oncological clinical trials of MGN1703 already confirmed our design.

## Introduction

Oligodeoxynucleotides containing nonmethylated cytosine moieties within cytosine–guanine sequence motifs (CpG ODN) are potent immunomodulators [1,2]. Nonmethylated CG sequence motifs resemble bacterial, certain viral, and also mammalian mitochondrial DNA in terms of this signature [1–5] and are therefore recognized as “danger signals” if such DNA is detected in nonappropriate intracellular environments by the innate immune system. Nonmethylated CG motifs either signal infection as pathogen-associated molecular patterns or are interpreted as the molecular signature of decaying mammalian cells, then termed damage associated molecular patterns. CpG ODN bind and activate Toll-like receptor 9 (TLR-9), which in the human immune system is located in the endoplasmic reticulum of plasmacytoid dendritic cells (pDC) and B cells [6]. TLR-9 activation subsequently triggers a signaling cascade involving MyD88, IRAK, and TRAF-6 leading to the activation of NF–κB and IRF7 pathways [1,2,7–9]. This results in the production of proinflammatory cytokines and the type 1 interferon response, leading to the activation and maturation of dendritic cells (Langerhans cells, pDC, myeloid dendritic cells mDC), the proliferation of B cells, and to the proliferation and activation of innate effector cells [e.g., natural killer (NK) cells and invariant NKT cells] among others. Eventually, through the bridging function of mature, activated DC the adaptive immune system responds [10–12].

CpG ODN can be classified into three separate classes with different structural characteristics and differentially enhancing antigen-specific humoral and cellular immune responses: class A are strong inducers of interferon (IFN)-alpha from pDC but very poor B cell activators and class B are potent stimulators of B cell proliferation with poor induction of pDC IFN-alpha secretion, while class-C CpG ODN exhibit moderate properties from both class A and class B [13,14].

Being stimulators of the innate and adaptive immune system, CpG ODN have shown promising therapeutic potential as vaccine adjuvants [15–18], as mediators of protective immunity against certain infections [19,20], and as immune therapeutics against cancer [21–23].

The most extensively studied single-stranded CpG ODN has been the Class B, PF-3512676 (ProMune, CpG-7909 or ODN2006) [4]. Two phase 3 trials of PF-3512676 administered in combination with standard chemotherapy regimens as first-line treatment in advanced non-small-cell lung cancer have not shown improvement in median overall or progression-free survival [25,26]. However, patients receiving PF-3512676 exhibited a higher proportion of adverse events ≥grade 3 in both studies. Due to the lack of significantly improved efficacy and increased toxicity resulting in an unfavorable risk–benefit profile seen with PF-3512676, both trials were terminated early.

Phosphorothioate (PT) modifications of the DNA backbone, usually introduced to enhance stability against degradation *in vitro* and *in vivo* [27–29] rendered the resulting ODN-PT much more potent in stimulation of B-cell proliferation than the corresponding ODN with a natural phosphorodiester backbone (ODN-PO) [10,30]. However, PT-modifications result in several toxic side effects that may—at least in part—account for the above-mentioned clinical failures: CpG ODN-PT lead to a prolongation of the blood clotting time via inhibition of the intrinsic tenase complex [31–33]. CpG ODN-PT may affect cell signaling by nonspecific binding to various proteins (i.e., transcription factors) [4]. In rhesus monkeys CpG ODN-PT lead to acute toxicities via complement activation [35,36], an effect that also appeared after treatment of normal donor serum with CpG ODN-PT [3]. In rats, CpG ODN-PT were stronger promoters of thrombocytopenia, anemia, or neutropenia than the corresponding CpG ODN-PO [7]; and in mice, the PT-backbone showed a synergistic effect in the induction of arthritis [8]. Most importantly, however, CpG ODN-PT accounted for dramatic destruction and reduced functionality of lymphoid organs in mice [9].

PT or other modifications to protect against exonucleolytic degradation can be avoided if DNA molecules lack open ends like in covalently-closed dumbbell-like structures [40–42]. By this general design principle, we developed a new family of TLR-9 agonists by the name of double stem-loop immunomodulators (dSLIM^®^). dSLIM are synthesized by ligation of two identical DNA molecules with stable stem-loop structures, resulting in covalently closed molecules of natural DNA (i.e., with a 3′, 5′-phosphorodiester backbone), which descriptively form a dumbbell-like structure. The immunomodulatory nonmethylated CG motifs are placed in the single-stranded loops, double-stranded stem, or in both.

Here, we present the molecular design path to MGN1703 from other molecules of the dSLIM family. To assay the respective design variants for activation of the TLR-9 pathway, we first employed specific cell lines of different origin as a model system and later confirmed our data in human peripheral blood mononuclear cells (PBMC) from healthy donors. The cell line systems allowed for the detection of subtle differences in TLR-9 activation that would have been lost in PBMC assays due to the individual variability of PBMC from donors. On the other, only in PBMC most aspects of innate and adaptive immune activation by dSLIM molecules could be assessed. Finally, toxicological studies in rodents are reported.

## Materials and Methods

### TLR-9 agonists for immunomodulation

MGN1703 and other molecules of the dSLIM family were generated as described [43,44]. The endotoxin content of all nucleotide molecules was determined by an endpoint limulus amebocyte lysate test to be below 10 EU/mg DNA. If not denoted otherwise, a final concentration of 1 μM was used for each molecule.

To evaluate the effect of backbone modifications or substitution of CG motifs, we designed several variations (Mod-1 to Mod-9) of the standard MGN1703, a dumbbell-shaped molecule with two single-stranded loops of 30 nucleotides each containing three nonmethylated CG motifs and a 28-bp double-stranded stem. CG motifs were replaced by TG motifs to investigate CG dependence in control molecules (noCpG ODN, noCG-MGN1703).

### Cells

RPMI-8226, Jurkat, MOLT-3, U937, NK-92, and YT cells were obtained from the Deutsche Sammlung von Mikroorganismen und Zellkulturen GmbH (Braunschweig, Germany) and were cultured as recommended. Cells were stimulated with 1 μM of respective molecules of the dSLIM family or CpG ODN for 48 hours unless noted otherwise. Human peripheral mononuclear blood cells were isolated from peripheral blood of normal volunteers via Ficoll technique as described previously [45,46].

### Flow cytometry

Cells were stimulated with MGN1703 and other molecules of the dSLIM family and flow cytometry was performed as described [43,47]. Briefly, 48 hours after stimulation, cells were washed with phosphate-buffered saline (PBS) and 1×10^6^ cells were resuspended and stained with anti-CD80-fluorescein isothiocyanate (FITC), anti-CD86-phycoerythrin (PE), anti-CD40-FITC, anti-CD69-PE, anti-CD54-FITC, anti-human leukocyte antigen DR (HLA-DR)-PE, anti-CD28-FITC, anti-cytotoxic T-lymphocyte-associated protein 4 (CTLA4)-PE (all from Becton Dickinson), or anti-human leukocyte antigen ABC (HLA-ABC)-PE (Dako Cytomation) and incubated for 30 minutes. The cells were subsequently washed with PBS [Dulbecco's PBS 0.0095 M (PO4) w/o calcium and magnesium, Lonza]. The isotypes immunoglobin G (IgG)1-FITC, IgG2a-FITC, IgG2a-PE, and IgG1-PE (all from Becton Dickinson) were always used as negative controls. Necrotic cells were detected by propidium iodide staining and excluded from analysis. The measurements were carried out using a FACScalibur flow cytometer (Becton Dickinson) and analyzed via CellQuest and WINMDI2.8 Software.

### Enzyme-linked immuno assay

Cytokine enzyme-linked immuno assays were performed with supernatants of 10^6^ cells/mL that had been cultured for 48 hours according to the manufacturer's instruction [IFN-γ, interleukin (IL)-12p40, IL-6, and IFN-α, R&D Systems].

### Stability assay

A stability study according to International Conference on Harmonisation over 36 months at –20°C has been performed for MGN1703. Identity, purity, and content were analyzed via photometric determination, high-performance liquid chromatography (HPLC), and gel electrophoresis. This stability data fulfilled all set specifications.

### Toxicology studies in mice

Mice studies were performed as described previously [9]. Briefly, groups consisting of 6-week-old, female C57BL6 mice were administered PBS (control), MGN1703, and ODN1826 intraperitoneally at different doses (in 100 μL volume) once daily for 7 days. At the end, all mice were sacrificed and organs were weighed immediately. Subsequently, liver and spleen were stored in 4% paraformaldehyde at room temperature. Paraformaldehyde-fixed sections of spleen and liver were stained with hematoxilin/eosin for histological report. Analysis was performed as a double-blind evaluation. An analysis of variance test was used for statistical analysis of organ weights. Mice studies were performed in accredited animal facilities at Dirección General de Agricultura, Comunidad de Madrid (EX–023-USC) following the respective standard operating procedures, directives, and local laws: [European Directives 2003/65/EC/86/609/CEE; Spanish Law RD 223/1988; Humane Endpoints Guidance Document of the Organisation for Economic Co-operation and Development ENV/JM/MONO(2000)7; and Federation of Laboratory Animal Science Associations guidelines].

## Results

### MGN1703, a member of the dSLIM family

**FIG. 1.**
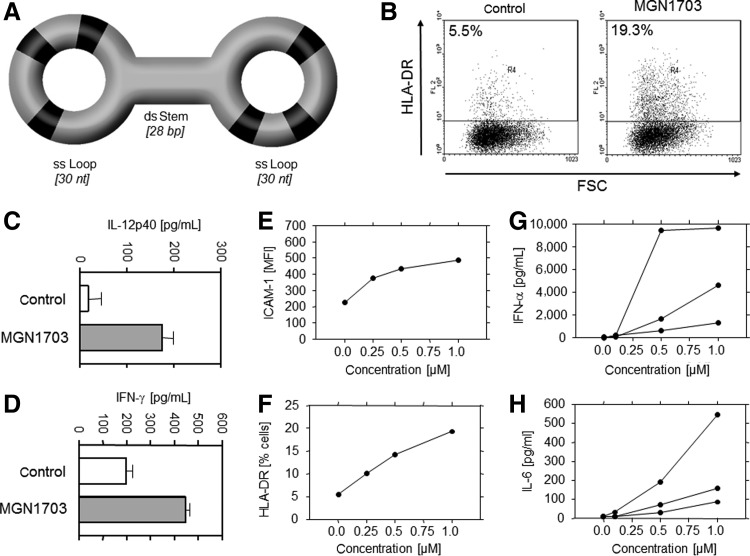
Immunomodulatory potential of MGN1703. **(A)** Schematic draw of a member of the double stem loop immunomodulators family (MGN1703) with cytosine–guanine (CG) motifs in *black*. ds, double strand; ss, single strand **(B)** Representative flow cytometric analysis of RPMI-8226 cells incubated with or without MGN1703 for 48 hours. FSC, forward scatter. **(C)** Interleukin (IL)-12p40 enzyme-linked immune assay (ELISA) of supernatant from RPMI-8226 cells. **(D)** Interferon-gamma (IFN-γ) ELISA of supernatant from natural killer (NK)-92 cells. **(E, F)** Example of a concentration-dependent stimulation of ICAM-1 and human leukocyte antigen (HLA)-DR surface expression on RPMI-8226 analyzed via flow cytometry. **(G, H)** Concentration-dependent activation of IFN-α and IL-6 cytokine production from healthy donor peripheral blood mononuclear cell (PBMC) analyzed via ELISA. Each line represents one donor.

Commonly used PT-modified CpG ODN are known for clinically relevant side effects, such as unspecific protein binding, prolongation of blood clotting, and acute toxicities [31–39]. As a radical alternative we designed a family of double stem-loop immunomodulatory DNA molecules (dSLIM) with natural phosphorodiester (PO) DNA backbones lacking open 3′ and 5′ ends. Such covalently closed constructs can be drawn as a dumbbell-like shape, where two single-stranded loops are connected through a double-stranded stem. One member of the dSLIM family, MGN1703, consists of a stem of 28 base pairs (bp) and two loops of 30 nucleotides (nt) with identical sequences, containing three nonmethylated CG motifs each (Fig. 1A). The MGN1703 sequence was confirmed by standard sequencing of the primary ODN before the ligation step. Integrity of the covalently closed structure was assayed via exonuclease digestion and subsequent gel electrophoresis or HPLC. MGN1703, when stored at a temperature of −20°C, was stable for a minimum of 36 months (data not shown).

### MGN1703 increases expression of immunological surface markers and cytokine secretion

CpG ODN are known to stimulate expression of immunological markers on the cell surface and production of cytokines *in vitro* [1,2]. We investigated the effect of MGN1703 on various cell lines representing components of innate and adaptive immune responses, like B-lineage cells (RPMI-8226), T-lineage cells (Jurkat, MOLT-3), monocytes (U937), and NK cells (NK-92, YT). Analyzing surface expression of immunologically relevant molecules CD80/B7-1, CD86/B7-2, CD40, CD40L/CD154, CD28, CTLA-4/CD152, HLA-DR, ICAM-1/CD54 and CD69 by flow cytometry, we observed no direct effect of MGN1703 on T-lineage cells, NK cells or monocytes. However, B-lineage RPMI-8226 cells showed an increased surface expression of CD80/B7-1, CD40 (Supplementary Fig. S1; Supplementary Data are available online at www.liebertpub.com/nat), HLA-DR (Fig. 1B), and ICAM-1/CD54. Regarding cytokine secretion, only a moderate stimulation of IL-12 (p40 subunit) production from RPMI-8226 (Fig. 1C) and IFN-γ secretion from NK-92 cells (Fig. 1D) was found after incubation with MGN1703, confirming that signaling and interaction between different cell types are required to generate the typical downstream effects of TLR-9 activation by MGN1703. In keeping with this, human PBMC, when incubated with MGN1703, induced production of a specific cytokine pattern consisting of T helper cell 1 (T_H_1) cytokines IFN-α, IFN-γ, and IL-12p40, and the proinflammatory cytokine IL-6 (Table 1). All these immunomodulatory effects were clearly dose dependent (Fig. 1E–H).

**Table 1.** Stimulation of Human Peripheral Blood Mononuclear Cells with MGN1703

	*IFN-α [pg/mL]*	*IL-6 [pg/mL]*	*IFN-γ [pg/mL]*	*IL-12p40 [pg/mL]*	*IL-2 [pg/mL]*	*TNFα [pg/mL]*
Control	4	<25^a^	<15^a^	<25^a^	46±25	16±1
MGN1703	1712±342	567±456	116±40	117±25	101±36	52±3

Human peripheral blood mononuclear cells were incubated with 1 μM MGN1703 for 48 hours and cytokine levels analyzed via enzyme-linked immune assay.

Results presented as mean values±standard error.

^a^ Below the detection level of the enzyme-linked immune assay.

IFN, interferon; IL, interleukin; TNF, tumor necrosis factor.

### Immunomodulatory potential of MGN1703 is strictly structure dependent

**FIG. 2.**
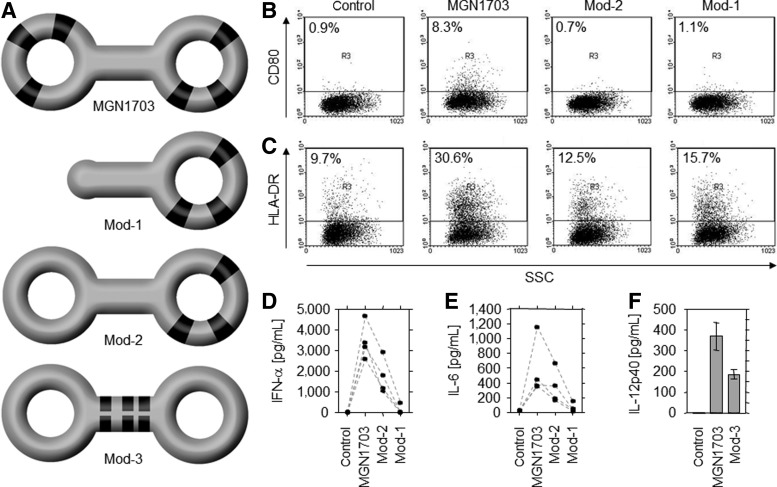
Influence of structure and CG motif availability on function of MGN1703. **(A)** Schematic drawing of MGN1703 and modified molecules with CG motifs in *black*. **(B, C)** Stimulation of CD80/B7-1 **(B)** and HLA-DR **(C)** surface expression on RPMI-8226 cells analyzed via flow cytometry. SSC, side scatter. **(D, E)** Activation of normal donor PBMC to produce IFN-α **(D)** and IL-6 **(E)**, which was analyzed via ELISA. *Dotted lines* connect the samples from one donor. **(F)** Activation of IL-12p40 production by murine spleen cells as analyzed by ELISA.

Some reports described the requirement of nucleic acid secondary structure or the buildup of higher molecular complexes as a prerequisite of immunological efficacy [48,49]. Therefore, we designed variants of stem-loop constructs differing in number and distribution of CG motifs, stem and loop size, and number of loops and evaluated their modulatory potential. Besides MGN1703, these were a single-loop stem construct (Mod-1), a double-loop stem construct with CG motifs in only one loop (Mod-2), and another double-loop stem construct with CG motifs in its stem (Mod-3) (Fig. 2A). All molecules contained the same immunomodulatory sequence, a stretch of at least 20 nucleotides containing 3 CG motifs. Regarding expression of surface molecules CD80/B7-1 and HLA-DR, a change in structure and number of CG motifs reduced the activation potential compared with MGN1703 (Fig. 2B, C).

**FIG. 3.**
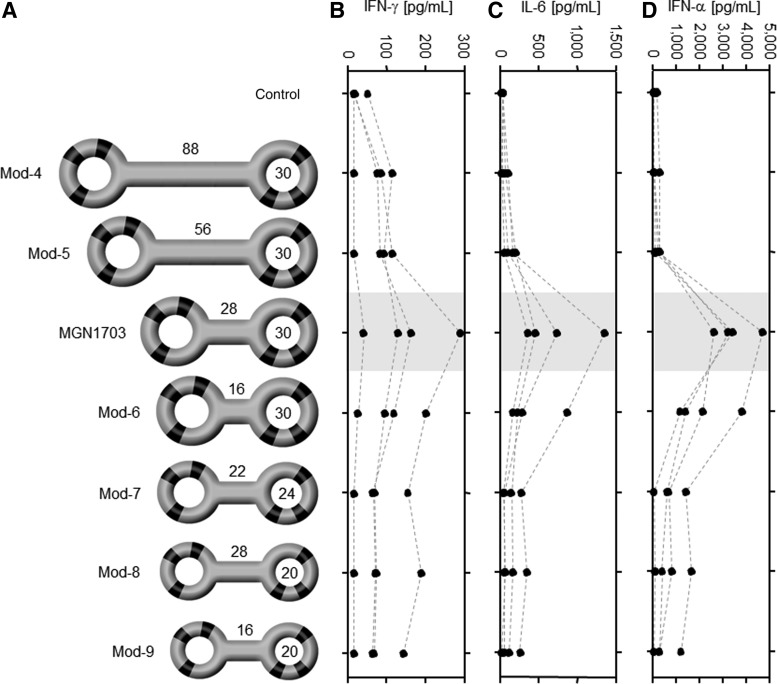
Influence of size on function of MGN1703. **(A)** Schematic drawings of MGN1703 and derived molecules with CG motifs shown in *black* (Mod-4 to Mod-9). Numbers of nucleotides are denoted in the *loops*; numbers of base pairs are shown on top of the stem. **(B–D)** Activation of healthy donor PBMC to produce IFN-γ **(B)**, IL-6 **(C)**, and IFN-α **(D)** which was analyzed via ELISA. *Dotted lines* connect the samples from one donor.

In keeping with this, MGN1703 also generated the highest stimulation of cytokine production compared with the molecules when IFN-α and IL-6 production from healthy donor PBMC and IL-12p40 from murine spleen cells were analyzed (Fig. 2D–F). To investigate the importance of structure in more detail, we generated further variations of MGN1703 with increased (from 28 bp to 56 bp and 88 bp) or decreased stem size (from 28 bp to 22 bp and 16 bp). Accordingly, we decreased the MGN1703 loop size (from 30 nt to 24 nt and 20 nt) while maintaining the 20-nt immunomodulatory sequence of that encompasses the CG motifs. The resulting molecules Mod-4 to Mod-9 are shown in Figure 3A. Notably, these structure variations of MGN1703 clearly diminished its immunomodulatory potential, as shown by IFN-α, IFN-γ, and IL-6 production from PBMC (Fig. 3B–D). Using even smaller constructs with only one CG motif per loop or two CG motifs in the stem confirmed the necessity of CG motifs in both loops but not in the stem for a high potency (Supplementary Fig. S2). These data clearly demonstrate the importance of a certain structure, size, CG motif content, and positioning of CG motifs on the efficacy of MGN1703 in both human and murine cells.

### MGN1703 does not induce toxicities in rodents

**FIG. 4.**
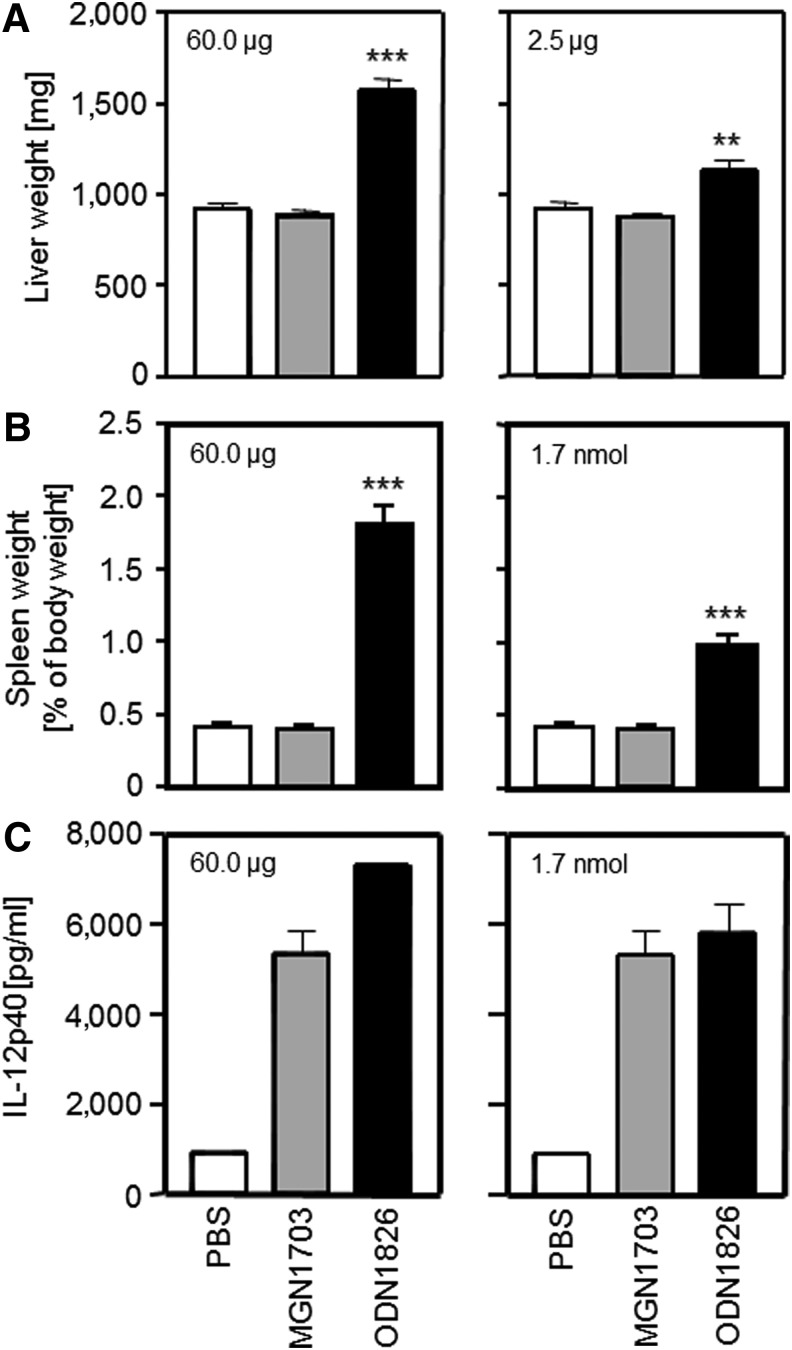
Effect of MGN1703 and phosphorothioate (PT)-based oligodeoxynucleotide 1826 (ODN1826) on liver and spleen size from mice and cytokine production *in vivo*. **(A)** Absolute liver weight after seven daily injections of each 60 μg (left) and 2.5 μg (right) of MGN1703 (*gray bars*) or ODN1826 (*black bars*). **(B)** Relative spleen weight after seven daily injections of equal mass (60 μg, left) and equimolar (1.7 nmol, right) amounts of MGN1703 (*gray bars*) or ODN1826 (*black bars*). **(C)** *In vivo* serum levels of IL-12p40 after seven daily injections of equal mass (60 μg, left) and equimolar (1.7 nmol, right) amounts of MGN1703 (*gray bars*) or ODN1826 (*black bars*). Statistically significant differences to the phosphate-buffered saline (PBS) control are denoted with stars (**p<0.01, ***p<0.001).

Heikenwalder et al. showed that a CpG ODN-PT resulted in significant toxic effects in mice (i.e., dramatically reduced functionality and definition of lymphoid organs) [9]. To confirm the safety of MGN1703, we used a similar mouse model and successive injections of 2.5 μg or 60 μg of DNA-based immunomodulators, comparing MGN1703 consisting of natural DNA entirely with ODN1826 as a CpG ODN with a PT-modified backbone. After seven daily intraperitoneal injections of MGN1703, we did not detect any increase of liver, spleen, or lymph node weight compared with PBS injection, neither with low dose (2.5 μg) nor high dose (60 μg) of MGN1703 (Fig. 4A, B).

**FIG. 5.**
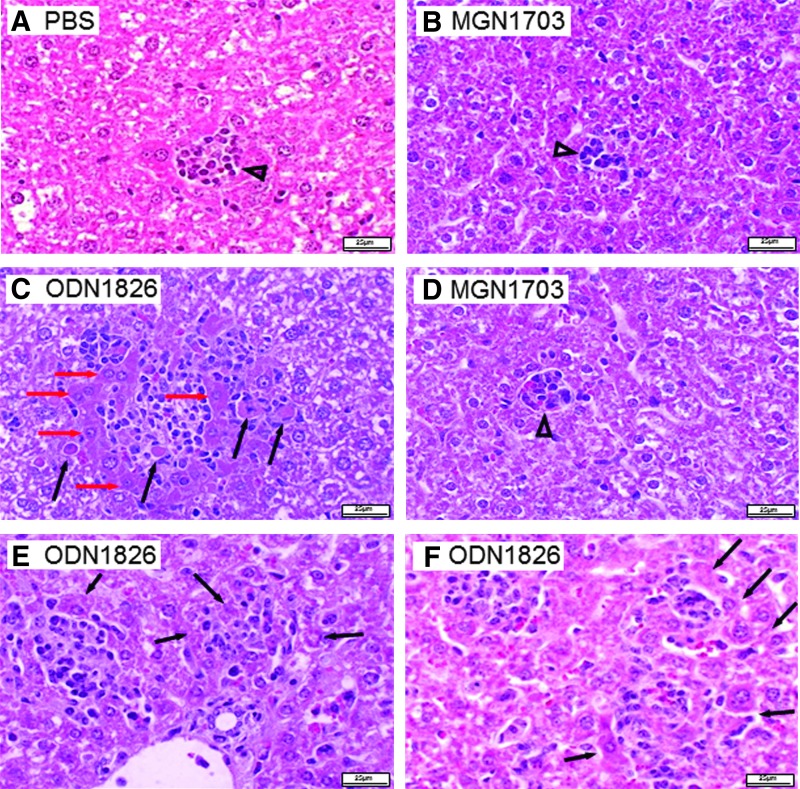
Histopathology of the liver after seven daily injections of MGN1703 or PT-based ODN1826 into mice. **(A)** Injection of PBS (control); **(B)** 7×2.5 μg MGN1703; **(C)** 7×2.5 μg ODN1826; **(D)** 7×60 μg MGN1703; **(E, F)** 7×60 μg ODN1826. **(A, B, D)** Minor inflammatory reaction (*triangles*) predominantly associated to the presence of macrophages and neutrophils. **(C)** Multiple inflammatory areas with necrotic hepatocytes (*black arrows*) and incipient necrosis (*red arrows*): intense presence of macrophages and some neutrophils. **(E, F)** Multiple inflammatory areas with incipient necrosis of hepatocytes (*black arrows*): intense eosinophilic response in the cytoplasm, nuclear pyknosis, and karyolysis. Representative pictures are shown.

**FIG. 6.**
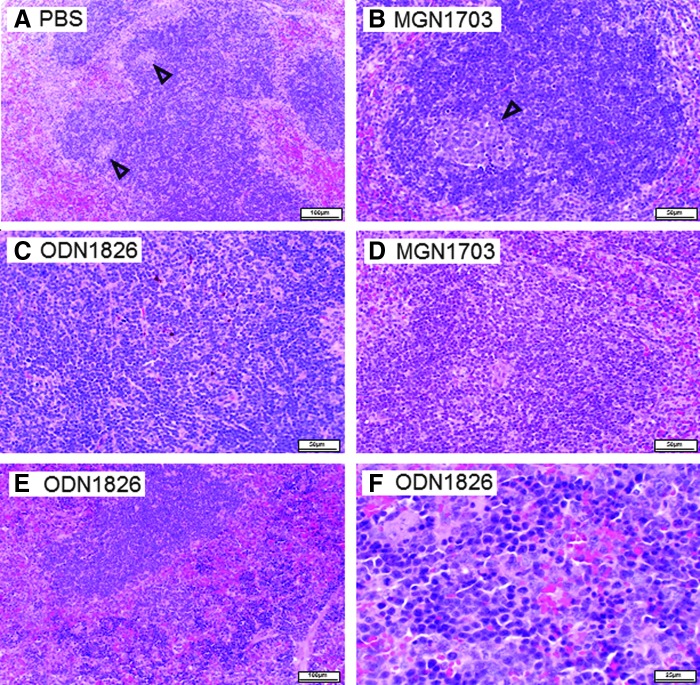
Histopathology of the spleen after seven daily injections of MGN1703 or PT-based ODN1826 into mice. **(A)** Injection of PBS (control); **(B)** 7×2.5 μg MGN1703; **(C)** 7×2.5 μg ODN1826; **(D)** 7×60 μg MGN1703; **(E, F)** 7×ODN1826. **(A, B, D–F)** White pulp shows a normal structure, including germinal centers (*triangles*). **(C)** Normal lymphoid cells; no evidence of follicular germinal centers. **(E)** Red pulp hyperplasia, showing macrophages, myelopoietic, and megacaryocytic cells with extensive histiocytic cells. **(F)** Red pulp: lymphoplasmocytic and hematopoietic cells with extensive histiocytic cells. Representative pictures are shown.

In contrast, ODN1826 led to significant weight increase of these organs—with the 2.5 μg dose and even more with 60 μg of the CpG ODN-PT (Fig. 4A, B). This repeated the experiments with and confirmed the high toxicity of the CpG ODN described in the paper by Heikenwalder et al. [9]. The absence of toxicity after MGN1703 injections and the drastic toxicity from ODN1826 injections was not due to the difference in molecular weight (MGN1703>ODN1826) and the resulting higher number of ODN1826 molecules compared with MGN1703 at equal masses of injected molecules. As shown in Figure 4C, the highly significant differences were also observed if equimolar amounts of the respective molecules were injected, though both MGN1703 and ODN1826 were able to raise IL-12p40 levels in mice sera (Fig. 4C). Histopathological analyses of liver and spleen tissues confirmed the differences as well (Figs. 5, 6). While MGN1703 had only a slight effect on histopathology, ODN1826 resulted in vastly increased liver damage from inflammatory reaction and hepatocyte necrosis (regarding number and size of lesions). Damage of spleen produced similar impressive evidence, such as red pulp hyperplasia with intense extramedullary hematopoiesis and alteration of the white pulp (Table 2). The organ alterations caused by ODN1826 administration are not the equivalent of a normal immune response but do indicate toxic effects on the tissues analyzed.

**Table 2.** Histopathological Evaluation of Liver and Spleen from Mice After Seven Injections of MGN1703 or Phosphorothioate-Based ODN1826

*Histopathology*	*Control*	*MGN1703 [60 μg/day]*	*ODN1826 [60 μg/day]*	*MGN1703 [2.5 μg/day]*	*ODN1826 [2.5 μg/day]*
Liver damage					
Necrotic hepatocytes	(+)	(+)	+++	(+)	++
Inflammtory reaction	(+)	(+)	+++	(+)	++
Spleen damage					
Uniform lymphoid population	–	(+)	+	–	+
Diffuse lymphoid activation	–	–	+	–	+
White pulp hyperplasia	–	–	–	–	(+)
Red pulp hyperplasia	–	–	+++	–	–

(+)/+/++/+++, degree of changes in relation to ODN1826 [60 μg/day] as group with most severe changes; – not observed.

This led us to evaluate the safety of MGN1703 under the defined conditions of good laboratory practice (GLP). We performed acute toxicity studies in rats by single intravenous injections of 0.8, 2.7, or 10.4 mg/kg of MGN1703 or subcutaneous injections 0.8, 3.1, or 15.4 mg/kg. None of these injections resulted in any toxic effect, neither regarding mortality, clinical signs (i.e., changes in skin, fur, eyes, mucous membranes, occurrence of secretions and excretions, autonomic activity, presence of abnormal movements, etc.) nor significant alterations after macroscopic evaluation of the external surface of the body and necropsy of organs (kidneys, heart, spleen, liver, peritoneum, mesenteric lymph nodes, and thymus) (data not shown).

## Discussion

Backbone-modified ODN, especially CpG ODN-PT, have been evaluated in various clinical trials as promising therapeutics or adjuvants in the treatment of allergies, certain infections, and cancer [50,51]. However, they almost all failed in pivotal trials, probably due to a far too narrow therapeutic window. Safety concerns had earlier been raised from results of *in vivo* mice experiments, where commonly used CpG ODN-PT caused severe side effects such as destruction of lymphoid organs and hepatosplenomegaly [9].

As shown here, we have developed a new family of potent TLR-9 agonist—the dSLIM family of immunomodulators, with its most prominent member being MGN1703—consisting of only natural DNA to avoid toxicities and clinical side effects most likely being caused by the PT modifications, while maintaining specific immune activation properties. Protection against degradation by nucleases is achieved by the covalently closed molecular structure of MGN1703. We have shown that the immunomodulatory effect of MGN1703 is clearly dependent on its structure and the CG motif content as well. This fact is corroborated by experiments, where MGN1703 control molecules with no CG motifs had no relevant immunomodulatory effect (Supplementary Fig. S3).

Among the known side effects of CpG ODN-PT is the prolongation of blood clotting time [1]. As expected, the natural (PO) DNA of MGN1703 did not prolong activated partial thromboplastin time in peripheral blood samples from normal donors, whereas the CpG ODN-PT clearly did (Supplementary Fig. S4).

Selective CpG ODN have been characterized for optimal stimulation of murine, primate, or human cells [52,53]. Unlike PF-3512676 (ProMune, CpG-7909 or ODN2006), which was the TLR-9 agonist most often used in human clinical trials, MGN1703 overcomes such “species specificity,” facilitating the translation of mice experimental results into clinical trials. The efficacy of MGN1703, with its nonmodified DNA backbone, in both murine and human cells is perfectly in line with the results of Roberts and colleagues, who have shown that the species specificity of active CpG ODN is restricted to only CpG ODN-PT [4]. A synthetic CpG ODN analogue in which the deoxycytidine was replaced by a bicyclic heterobase also overcomes species specificity. The equivalent molecule but with a PT-modified backbone was rendered toxic and led to spleen enlargement in mice [5].

Secondary structure of MGN1703 as defined by size of double-stranded stem, size and number of loops, and distribution of the CG motifs is crucial for an optimal immunomodulatory potential. A possible explanation why stem-located CG motifs have significantly reduced immunomodulatory efficacy compared with CG motifs positioned in the loop can be derived from the work of Rutz et al. [9]. Binding studies suggest interaction of single-stranded, but not of double-stranded DNA with TLR-9 in a pH-dependent manner. Naturally occurring double-stranded DNA of bacteria is hypothesized to require the acidic conditions in the endosomes for degradation into multiple single-stranded CG motif fragments for subsequent signal transduction by TLR-9. In case of MGN1703, the CG motif located in the single-stranded loop is assessable and may directly interact with TLR-9, whereas in case of the modified molecule Mod-3 degradation of the double-stranded stem containing the CG motifs may be required, thus leading to decreased efficacy in TLR-9 activation.

There are discussions about the interaction of CG motif containing molecules with TLR-9 or with another molecule: On one hand, direct binding of single-stranded nonmethylated CpG ODN-PO to TLR-9 in a sequence- and pH-dependent manner has been demonstrated, and a possible DNA binding region within TLR-9 has been identified [9]. However, direct binding of PT ODN to TLR-9 occurred independent of CG motifs and was attributed to nonspecific binding of the PT backbone to proteins. Haas et al. showed that the sugar moieties of the DNA backbone determined recognition by TLR-9 [4]. PO-2′-deoxyribose homopolymers were found to bind and mediate activation of TLR-9. In contrast, PT-modified polymers bound TLR-9 100-fold more strongly, but competitively inhibited ligand-induced TLR-9 stimulation. CG motifs were able to convert this inhibition into strong TLR-9 activation. Accordingly, we hypothesize that MGN1703 containing only natural PO bonds in its DNA backbone mediates TLR-9 activation via its sugar backbone with CG motifs enhancing its immunomodulatory effects.

The requirement for TLR-9 in MGN1703-induced immunomodulation is corroborated by application of chloroquine, an inhibitor of endosomal acidification – a process necessary for TLR-9 signaling [49,56]. Chloroquine completely abrogated the induction of IFN-gamma-inducible protein 10 in MGN1703-stimulated PBMC and strongly diminished the percentage of activated enhanced green fluorescent protein–positive RAW264.7 reporter cells (transfected with human NF–κB responsive endothelial leukocyte adhesion molecule (ELAM) promoter–green fluorescent protein construct: ELAM 9) (Supplementary Fig. S5) [7]. Another indication for the importance of TLR-9 in the immunomodulation by MGN1703 is the inhibition of the stimulation by ODN with no CG motif (control molecule)—most likely due to competition at the recognition sites of TLR-9. Both, stimulation of surface marker expression as well as cytokine production were clearly reduced when MGN1703 and noCpG ODN were combined for incubation of PBMC or ELAM 9 cells (Supplementary Fig. S3 and Fig. S6). Furthermore, MGN1703 activated enriched pDC but not mDC to produce IFN-α (data not shown); pDC are known to be TLR-9 positive, whereas mDC do not express TLR-9.

TLR-9 has been found to be constitutively expressed as an inactive dimer and to change into an active confirmation upon binding of CpG DNA in the endosome so that the cytoplasmic TLR-9 Toll–interleukin 1 receptor signaling domains come in close proximity, enabling signal transduction by recruitment of MyD88 [8]. The specific secondary structure requirements for the optimally active MGN1703 molecule might thus result from a better induction of the conformational change of TLR-9.

On the other hand, a third molecule might hold the two TLR-9 receptors together and could also be involved in TLR-9 activation by holding two TLR-9 molecules together. A possible candidate is heat shock protein 90 (hsp90), which directly binds to CG motifs in CpG ODN-PO [9]. The importance of hsp90 for CpG ODN-PO–mediated signal transduction is also suggested by Okuya et al., who recently showed that hsp90 converted inert self-DNA or mainly CpG ODN-PO into potent triggers of IFN-α secretion [0]. Thus, the importance of secondary structure of MGN1703 might also result from different affinity to hsp90, or yet another unknown molecule involved in CG motif recognition and cellular or endosomal uptake or translocation.

In our mice experiments, the dose of MGN1703 for the toxicological studies was 3 mg/kg and 0.1 mg/kg body weight, adopted from Heikenwalder et al. [9]. Even though the dose of 3 mg/kg is considerably higher than the amount actually used of MGN1703 or CpG ODN in human clinical trials, the dose of 0.1mg/kg is at the lower end of the general human therapeutic range (0.1–0.6 mg/kg). Still, the low dose of PT-containing ODN1826 showed considerable toxicity in mice, whereas this was not observed with MGN1703. Together with the results of acute toxicity studies from GLP safety, we conclude that an application of MGN1703 should be safe, while exhibiting great immunomodulatory potential.

Consisting only of natural DNA, MGN1703 could better mimic the immunomodulatory effects of bacterial DNA, though avoiding the unwanted side effects of CpG ODN-PT. Thus, the dSLIM family of TLR-9 agonists—with its most prominent member being MGN1703—constitute a new class of efficacious immunomodulatory molecules free from any chemical modifications. In fact, MGN1703 has shown a good safety profile in a phase 1 clinical trial in patients with solid tumors [1]. Furthermore, it is currently being evaluated in clinical trials for the treatment of a variety of cancers. One, a randomized phase 2 trial in patients with metastatic colorectal carcinoma, has recently been completed and shown that MGN1703 maintenance treatment after first-line chemotherapy was well tolerated and appeared to induce durable and prolonged progression free survival and disease control in a subgroup of patients [2].

## Supplementary Material

Supplemental data

Supplemental data

Supplemental data

Supplemental data

Supplemental data

Supplemental data
