# Supplementary material for: Design and Structural Requirements of the Potent and Safe TLR-9 Agonistic Immunomodulator MGN1703
Source: Nucleic Acid Ther. 2015 Jun 1;25(3):130–40. doi: 10.1089/nat.2015.0533 (PMC4440985; doi:10.1089/nat.2015.0533)
Supplement: Supplemental data [file Supp_Figure1.pdf]

Supplementary Data

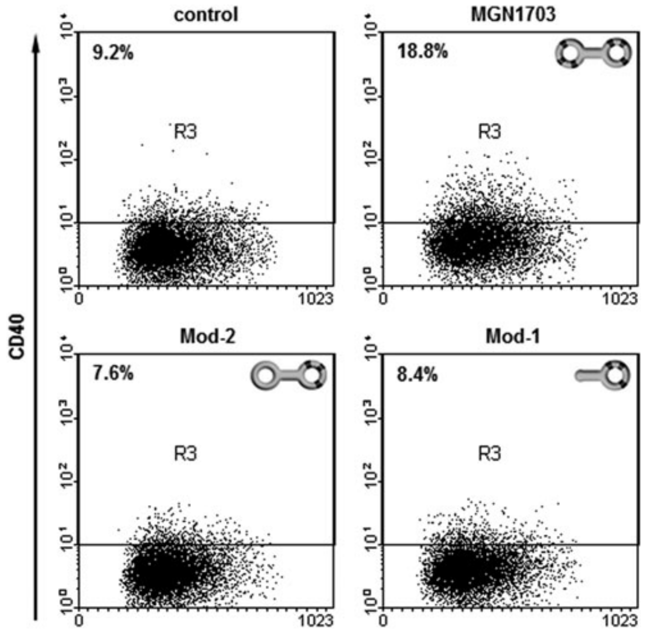

SUPPLEMENTARY FIG. S1. Representative flow cytometric analysis of CD40 on RPMI-8226 B-cells after incubation with MGN1703 and two modified members of the double stem-loop immunomodulators (dSLIM) family (Mod-1, Mod-2) compared with untreated cells (*upper left*).
