# Supplementary material for: Design and Structural Requirements of the Potent and Safe TLR-9 Agonistic Immunomodulator MGN1703
Source: Nucleic Acid Ther. 2015 Jun 1;25(3):130–40. doi: 10.1089/nat.2015.0533 (PMC4440985; doi:10.1089/nat.2015.0533)
Supplement: Supplemental data [file Supp_Figure2.pdf]

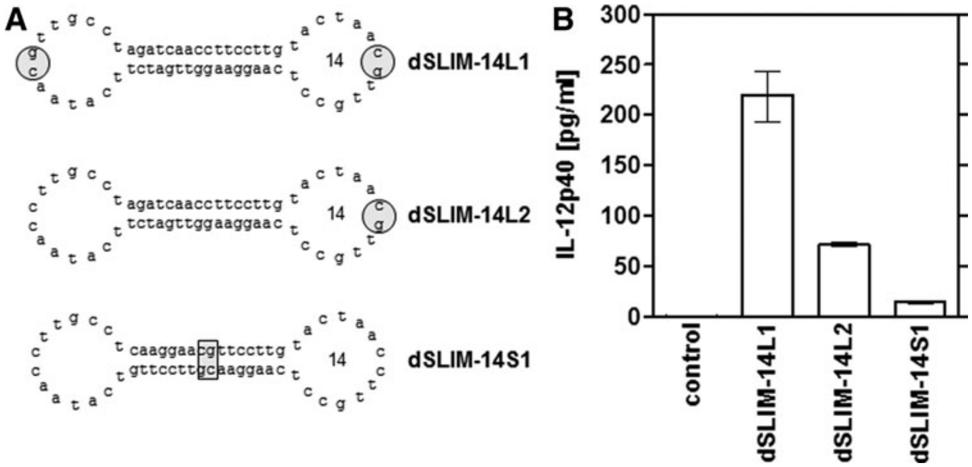

SUPPLEMENTARY FIG. S2. Influence of structure and cytosine–guanine (CG) motif availability on the function of dSLIM family members. **(A)** Schematic drawing of dSLIM-14L1 with one CG motif in each loop, dSLIM-14L2 with a CG motif in only one loop, and dSLIM-12S1 with a CG motif on the double-stem; CG motifs are *circled*. **(B)** Activation of interleukin (IL)-12p40 production by murine spleen cells as analyzed by enzyme-linked immuno assays (ELISA).
