# Supplementary material for: Design and Structural Requirements of the Potent and Safe TLR-9 Agonistic Immunomodulator MGN1703
Source: Nucleic Acid Ther. 2015 Jun 1;25(3):130–40. doi: 10.1089/nat.2015.0533 (PMC4440985; doi:10.1089/nat.2015.0533)
Supplement: Supplemental data [file Supp_Figure3.pdf]

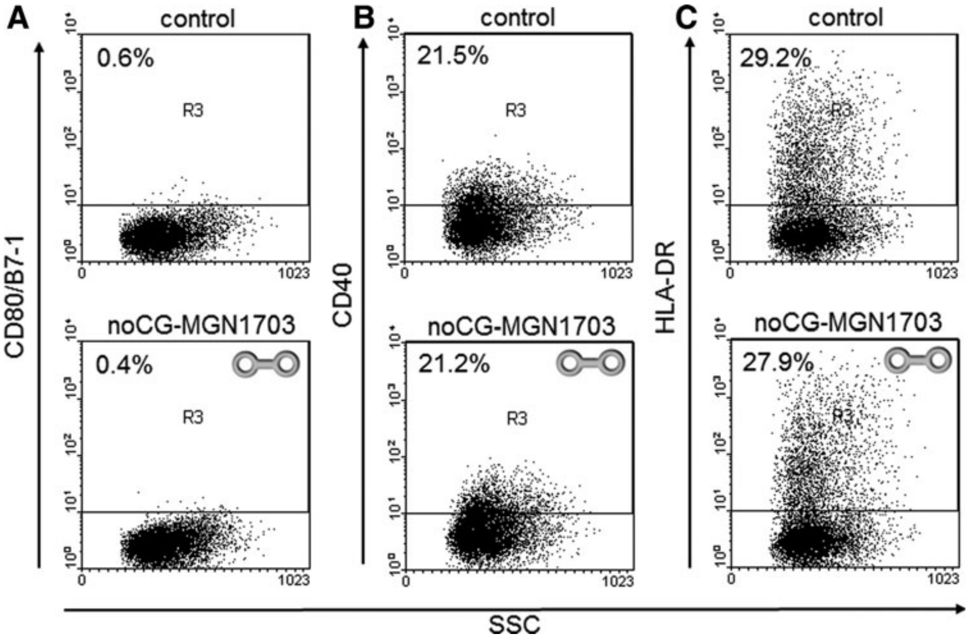

SUPPLEMENTARY FIG. S3. Analysis of MGN1703 molecules without CG motifs. Incubation of RPMI-8226 cells with noCG-MGN1703 (MGN1703 with no CG motif). Representative flow cytometric analysis after incubation with 1 μM noCG-MGN1703 for 48 hours (*bottom*) or untreated cells (*top*). **(A)** CD80/B7-1; **(B)** CD40; **(C)** HLA-DR. HLA, human leukocyte antigen.
