# Supplementary material for: Design and Structural Requirements of the Potent and Safe TLR-9 Agonistic Immunomodulator MGN1703
Source: Nucleic Acid Ther. 2015 Jun 1;25(3):130–40. doi: 10.1089/nat.2015.0533 (PMC4440985; doi:10.1089/nat.2015.0533)
Supplement: Supplemental data [file Supp_Figure4.pdf]

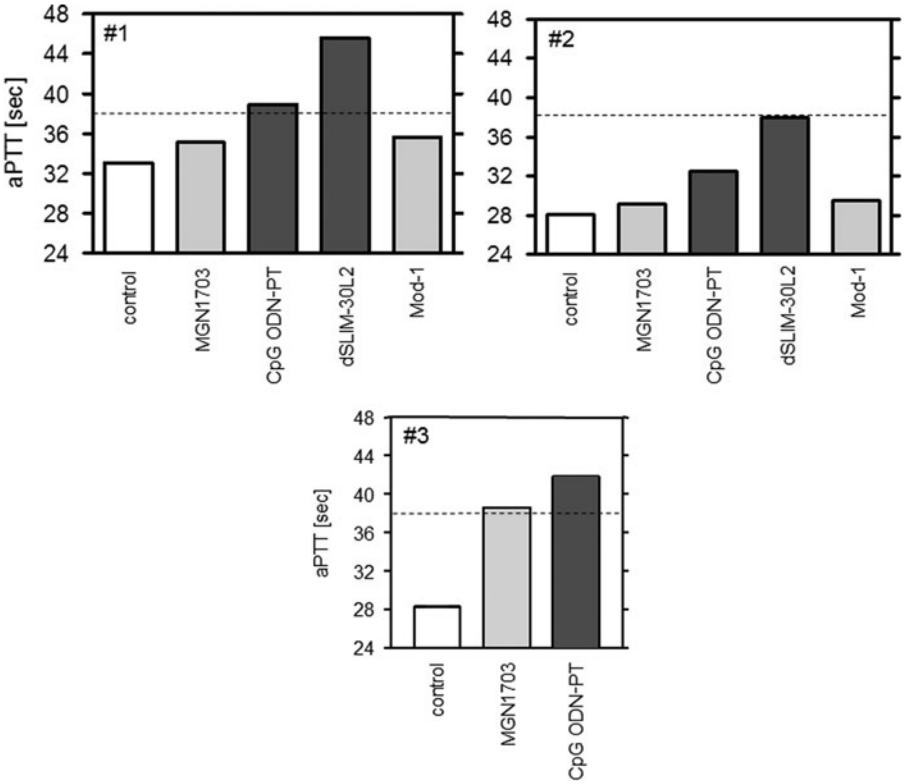

SUPPLEMENTARY FIG. S4. Influence of phosphorothioate (PT) content on activated partial thromboplastin time (aPTT) in PBMC from healthy donors. Incubation of peripheral blood mononuclear cells (PBMC) from three healthy donors (#1–#3) with MGN1703 or PT-based oligodeoxynucleotides containing nonmethylated cytosine–guanine motifs (CpG ODN-PT) with a similar modulatory sequence as MGN1703. PBMC from two donors (#1, #2) were also incubated with PT-based dSLIM-30L2, and Mod-1 (phosphorodiester-based dSLIM with one loop), showing the influence of PT but not structure/number of CG motifs on aPTT.
