# Supplementary material for: Design and Structural Requirements of the Potent and Safe TLR-9 Agonistic Immunomodulator MGN1703
Source: Nucleic Acid Ther. 2015 Jun 1;25(3):130–40. doi: 10.1089/nat.2015.0533 (PMC4440985; doi:10.1089/nat.2015.0533)
Supplement: Supplemental data [file Supp_Figure5.pdf]

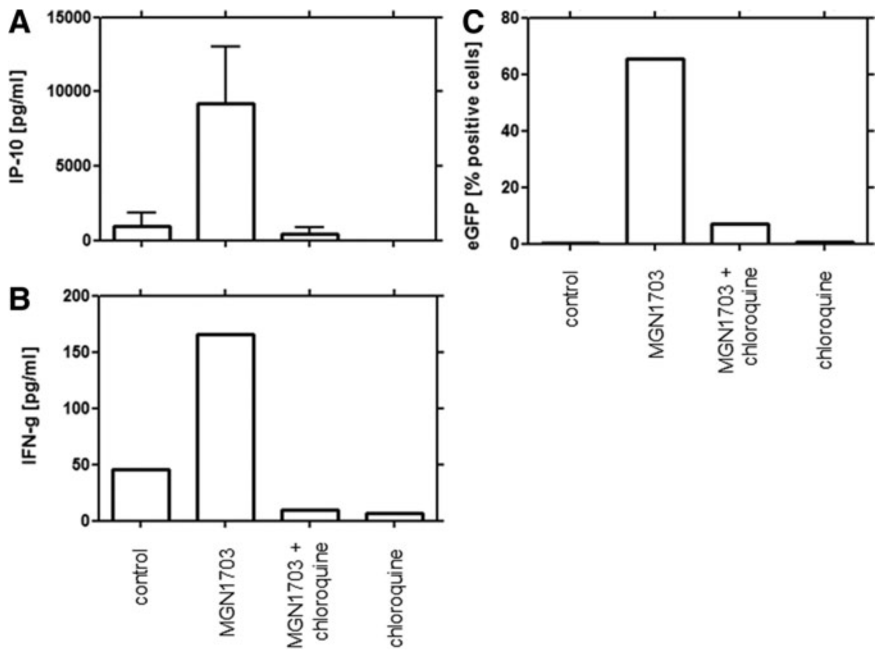

SUPPLEMENTARY FIG. S5. Influence of chloroquine on immunomodulation by MGN1703. **(A, B)** Incubation of PBMC from healthy donors with MGN1703 with or without chloroquine: IP-10 **(A)** and interferon gamma (IFN-γ) **(B)** as analyzed by ELISA. **(C)** Analysis of enhanced green fluorescent protein–positive RAW264.7 reporter cells (transfected with human NF-κB–responsive endothelial leukocyte adhesion molecule (ELAM) promoter–green fluorescent protein construct ELAM 9) after incubation with MGN1703.
