# Supplementary material for: Design and Structural Requirements of the Potent and Safe TLR-9 Agonistic Immunomodulator MGN1703
Source: Nucleic Acid Ther. 2015 Jun 1;25(3):130–40. doi: 10.1089/nat.2015.0533 (PMC4440985; doi:10.1089/nat.2015.0533)
Supplement: Supplemental data [file Supp_Figure6.pdf]

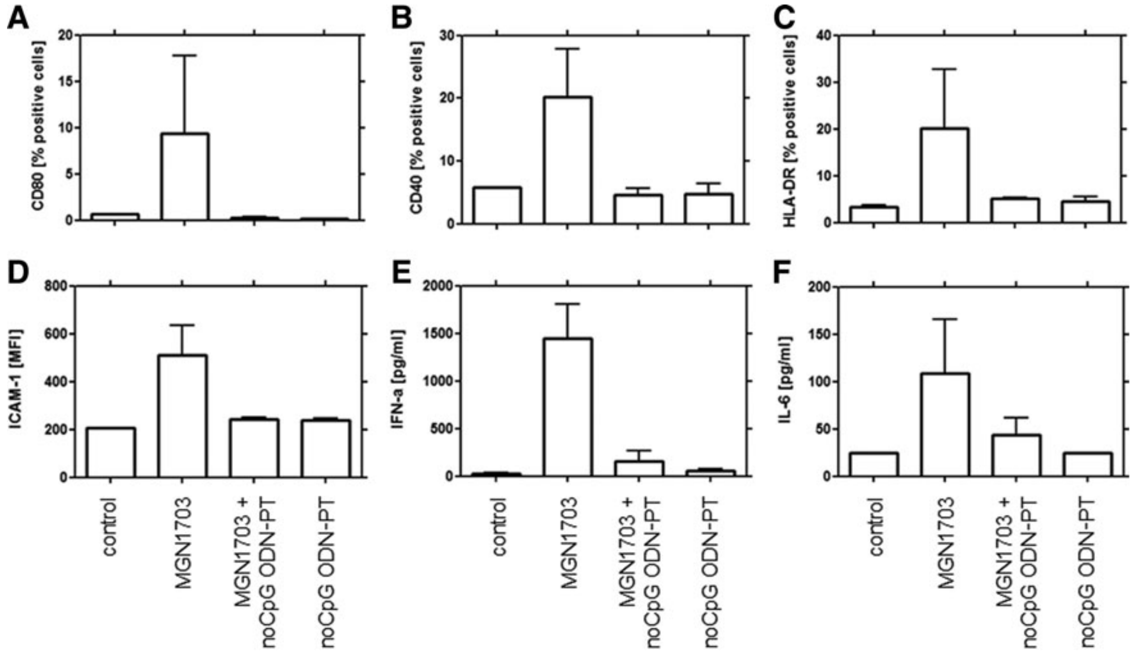

SUPPLEMENTARY FIG. S6. Influence of co-incubation with a noCpG ODN-PT (ODN-PT with no CG motif) with a similar sequence. **(A–D)** Incubation of RPMI-8226 B cells and subsequent flow cytometric analysis: **(A)** CD80/B7-1; **(B)** CD40; **(C)** HLA-DR; **(D)** ICAM-1. **(E, F)**, Incubation of PBMC from healthy donors and subsequent ELISA for IFN-α **(E)** and IL-6 **(F)**.
